# Serological and Molecular Investigation of Infectious Laryngotracheitis Virus in Chickens from Robe Town, Southeastern Ethiopia

**DOI:** 10.3390/ani14223227

**Published:** 2024-11-11

**Authors:** Samuel Abebe, Gianmarco Ferrara, Belayneh Getachew, Eyob Hirpa, Nebyou Moje

**Affiliations:** 1 Faculty of Veterinary Medicine, Hawassa University, Hawassa P.O. Box 05, Ethiopia; edegdvm2016@gmail.com; 2 Department of Veterinary Science, University of Messina, 98166 Messina, Italy; gianmarco.ferrara@unina.it; 3 National Veterinary Institute (NVI), Bishoftu P.O. Box 19, Ethiopia; belaynehgetachew@gmail.com; 4 College of Veterinary Medicine and Agriculture, Addis Ababa University, Bishoftu P.O. Box 34, Ethiopia; eyob.hirpa@aau.edu.et

**Keywords:** avian infectious laryngotracheitis, ILTV, infectious laryngotracheitis virus, molecular characterization, seroprevalence, risk factors, Southeastern Ethiopia

## Abstract

Infectious laryngotracheitis virus (ILTV) is responsible for avian infectious laryngotracheitis (ILT), a highly contagious acute respiratory disease affecting chickens. However, there is limited information on ILTV and its distribution in Ethiopia, particularly in the southeastern region. The aim of this study was to establish the serological prevalence and molecular evidence in commercial and backyard chickens from Robe town, Southeastern Ethiopia. A cross-sectional study was conducted between December 2021 and June 2022, collecting 240 serum samples from randomly selected chickens belonging to eight kebeles (farms) using systematic random sampling. ILTV-specific antibodies were detected using a commercial indirect enzyme-linked immunosorbent assay (ELISA). From 240 serum samples, 26.7% were positive for ILTV antibodies. Logistic regression analysis identified the type of poultry farm (backyard) and the introduction of chickens from other farms as potential risk factors associated with ILTV exposure. Tracheal tissue and oropharyngeal and tracheal swabs were collected from suspected chickens for isolation and molecular detection. A total of six samples were successfully isolated in embryonated eggs (40%), with four of them verified with a specific PCR. These findings documented the presence of ILTV in the study area, which needs further insight to fully understand the actual spread of ILTV and quantify the damage caused to the poultry sector.

## Simple Summary

Infectious laryngotracheitis virus (ILTV) causes avian infectious laryngotracheitis (ILT), a highly contagious acute respiratory disease that affects chickens. This infection is ubiquitous globally and decreases poultry production, with significantly more catastrophic repercussions in states with unstable economic systems. In this study, a total of 240 sera from eight farms (including commercial and backyard) were sampled to evaluate ILTV exposure. A total of 64 samples tested positive by commercial ELISA. The risk analysis identified higher prevalences in backyard chickens and farms that introduced new animals from other farms. Furthermore, 15 suspect animals were sampled for the viral isolation in embryonated eggs. Isolation was successful in six samples, of which four were confirmed by PCR using specific primers. This study highlights the presence of this virus in different types of poultry farms in Southeastern Ethiopia and identifies some management practices that favor the spread of this infection.

## 1. Introduction

Chicken production is widely spread in Ethiopia and represents a valuable source of protein and income, especially for rural areas [1]. About 97% of the Ethiopian poultry population consists of indigenous chickens, while the remaining 3% consists of imported exotic and hybrid breeds of chickens [2]. The consequences of globalization, climate change, and the rapidly expanding poultry population favor the emergence of several diseases [3]. Among these emerging diseases, avian infectious laryngotracheitis (ILT) is an acute, highly transmissible viral disease of chickens, mainly affecting the upper respiratory tract. The etiological agent is *Gallid alphaherpesvirus* type 1 (GaHV-1), which belongs to the genus Iltovirus, family Herpesviridae, and subfamily Alphaherpesvirinae [4,5]. The virus has a linear double-stranded DNA genome of approximately 155 kb that encodes 80 viral proteins [6]. These envelope proteins, including gB, gC, gD, gG, gH, gJ, gM, and gN, are glycosylated, and they are deputies of several functions, such as the mediation of attachment and entry into the host cell and the interaction with the host immune system [7,8].

Although chickens are the primary host, occasional infections of pheasants, partridges, and peafowl have been reported, while several species, including starlings, sparrows, crows, pigeons, and ducks, seem to be resistant to the virus [3,9]. The virus is horizontally transmitted, and the primary replication site is the tracheal mucosa [10]. The outcome of infection depends on the virulence of the strain or co-infection with other respiratory pathogens (avian influenza virus (AIV), Newcastle disease virus (NDV), and infectious bronchitis virus (IBV)), with mortality rates ranging from 5% to 70% [11,12,13,14]. Two distinct clinical presentations are reported (severe and milder forms). The severe form causes significant dyspnea, expectoration of bloody mucus, and sneezing [3,4]. The milder form is associated with conjunctivitis, mucoid tracheitis, sinusitis, swollen infraorbital (almond-shaped eyes), nasal discharge, reduced egg production, poor weight gain, and low mortality [3,15]. GaHV-1, like other members of the herpes virus family, induces latent infections due to its persistence in the trigeminal ganglion of the central nervous system after 7 days of acute infection (which can reactivate under stress conditions) [15,16].

A laboratory diagnosis is required for ILT since other diseases cause similar clinical signs and lesions. Several methods can be carried out to confirm ILTV, including histopathology to detect syncytia and intranuclear inclusion bodies (INIBs), virus isolation, antigen detection with immunofluorescent antibodies (IFA) or immunohistochemistry (IHC), enzyme-linked immunosorbent assay (ELISA), direct electron microscopy (DEM), and DNA detection methods [17]. Furthermore, methods have developed rapidly in recent years. These can identify ILTV quickly and accurately, are highly sensitive, and successfully identify ILTV in clinical samples including the trachea, larynx, and conjunctiva [18,19].

Knowledge about the spread of this pathogen is fundamental in areas such as Ethiopia, where subsistence farming is also practiced. The first report of ILTV was reported by Mekibib et al. [4] from the southern part of Ethiopia. Furthermore, while other reports exist [20,21,22,23], there is a critical lack of information in Southeastern Ethiopia. The aim of this study was to determine the seroprevalence of ILTV in commercial and backyard farms from Robe town, Southeastern Ethiopia, and evaluate the potential risk factors involved in the spread of this infection. A second aim of this study was to attempt viral isolation to establish the molecular prevalence and strain for future investigations.

## 2. Materials and Methods

### 2.1. Description of the Study Area

**Figure 1:**
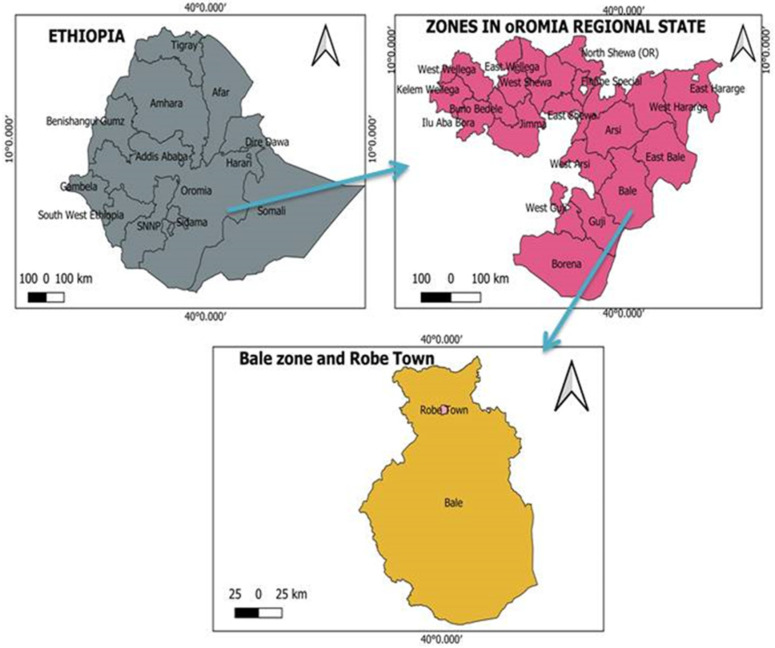
Map of the study district (Developed by QGIS).

The study was conducted in Robe Town, West Bale zone, Oromia regional state, Southeastern Ethiopia, from December 2021 to June 2022 (Figure 1). The area has an average annual temperature and humidity of 16.5 °C and 64%, respectively. The agro-climatic condition of the area is highland. In the area, there are two rainy seasons: the first and main season extends from August to December, and the second and shorter rainy season is from April to July. The dry season covers December to March [24]. In the study area, a total of 152,189 poultry are raised [25]. Robe Town and its surroundings were chosen because of the presence of traditional small poultry farms as well as the import of several exotic breeds of poultry from Central Ethiopia to the area, which may have contributed to the emergence of ILT.

### 2.2. Study Design, Study Population, and Sampling

A cross-sectional study was conducted from December 2021 to June 2022. The study populations included indigenous and exotic breeds of chickens reared in eight backyard and commercial farms in Robe Town. The chickens included in the study were healthy (for serological analysis) or diseased (for the viral isolation attempts), greater than 8 weeks old, and of both sexes. No commercial ILTV vaccine was available, and no vaccination programs had been implemented in Ethiopia. A multistage sampling was implemented to select the study zone, and a systematic random sampling technique was employed to select the village, flocks, and the number of chickens to sample from each farm. Purposive sampling through the evaluation of health status by clinical examination was employed to select diseased chickens (dyspnea, expectoration of bloody mucus, sneezing, high mortality, conjunctivitis, sinusitis, swollen infraorbital sinuses, nasal discharge, reduced egg production, poor weight gain) for isolation and molecular detection of ILTV. The sample size required for the seroprevalence study was determined based on sample size determination in random sampling for an infinite population with an expected prevalence of 19.4% [21] and with a confidence level of 95% and 5% desired absolute precision [26]. A total of 240 sera were collected from commercial and backyard chickens.

$$n=\frac{1.96^{2}+Pexp\left(1-Pexp\right)}{d^{2}}=\frac{1.96^{2}+0.194\left(1-0.194\right)}{0.05^{2}}=240$$

where n = sample size; P_exp_ = expected prevalence; and d = desired absolute precision.

The equal interval estimation during systematic random sampling was described as follows:

jth = N/n

where N indicates the total population of poultry and n indicates the sample size:

jth = N/n = 152,189/240 = 12th

Since maternal immunity is expected to develop during the first three weeks of life, chickens less than three weeks of age were excluded from the study. Each sample was collected aseptically from the wing vein (about 2–3 mL of blood) using a sterile syringe with 21-gauge needles and a vacutainer tube. Blood samples were immediately transported to the laboratory, centrifuged, and stored at −20 °C before being processed. Clinical samples (tracheal/oropharyngeal swabs and tracheal tissue) were taken from ILT-suspected chickens, inserted into cryovial tubes containing a virus transport medium supplemented with antibiotics, transported to the laboratory, and stored at −80 °C until the laboratory analysis was performed. Each sample was accompanied by a questionnaire with relevant information related to each chicken, including location, age, breed, sex, production type, and rearing method.

All chickens were sampled according to international animal care and use guidelines adopted by the Research Ethical Committee (ARSEC) of NVI [27]. Ethical clearance for the study was provided by the Research Ethics Committee of the Faculty of Veterinary Medicine, Hawassa University (FVM, HU). The research ethics committee of the FVM-HU reviewed and discussed this research on 19 September 2021 (Reference No. 620/w, date 7 July 2022).

### 2.3. Laboratory Analysis

#### 2.3.1. Serological Analysis

Serological tests were performed by an indirect commercial ELISA (ILTV Antibody Test Kit IDvet^®^ Screen^®^ ILT Indirect, 310 rue Louis Pasteur, Grabels, France) to measure specific antibodies against GaHV-1 in chicken sera. The test was performed according to the manufacturer’s instructions. Briefly, each test sample was diluted at 1:500 with sample diluents and incubated at room temperature for 60 min. After incubation, each well was washed three times with approximately 300 µL. After each wash, 100 µL of conjugate (anti-chicken IgG labeled with alkaline phosphatase) was added to each well and incubated at room temperature for 60 min. Following a further washing step, 100 µL of substrate reagent (TMB) was added to each well and incubated at room temperature for 15 min. Finally, 100 µL of stop solution (2M H_2_SO_4_) was added to each well to stop the reaction. The microtiter ELISA plate was placed in the ELISA reader to measure the OD (optical density) at 405 nm wavelength and interpret the results.

#### 2.3.2. Isolation and Molecular Detection

A total of 15 samples (6 oropharyngeal swabs and 9 tracheal swabs/tissues) were collected from suspected chickens for ILT virus isolation and molecular detection. Tracheal swabs/tissues were suspended in 10% (*w*/*v*) of sterile phosphate-buffered saline solution supplemented with penicillin and streptomycin (1000 μg/mL). The suspension was transferred into a sterile centrifuge tube and centrifuged at 3000 rpm for 10 min. The supernatant was harvested and employed for virus isolation and molecular detection. The tracheal tissue sample was chopped into small pieces using a sterile scalpel blade and minced using a mortar and pestle. The specimens were inoculated onto the chorioallantoic membranes (CAMs) of 10-day-old specific-pathogen-free (SPF) chicken embryos, which were incubated at 37 °C and examined daily for 5 days. CAMs and the allantoic fluids were harvested five days post-inoculation to collect the virus [28,29,30]. Briefly, embryonated SPF eggs were disinfected with 70% ethanol and inoculated with 0.2 mL of 10% of the supernatant using an insulin needle. Eggs were incubated at 37 °C and checked daily for embryo mortality. Any mortality within the first 24 h post-inoculation was considered non-specific, and the eggs were discarded. The dead embryo eggs were chilled at 4 °C for 24 h then opened aseptically, and the embryos were examined for gross ILT lesions [31]. The harvested allantoic fluid was added to sterile cryovial tubes and stored at −20 °C until DNA extraction was performed.

DNA extraction from the field sample tracheal/oropharyngeal swabs, tracheal tissue suspension, and allantoic fluid (n = 15) was performed with the QIAGEN DNA Mini Column Kit (QIAGEN, Frankfurt, Germany). Conventional PCR was used with a set of primers that specifically amplify a 688 bp fragment of the ICP4 gene. The PCR was conducted using Bio Rad 2729 Thermal Cycler (Hercules, CA, USA) in a reaction volume of 25 μL, containing 5 μL of 10× Dream Taq buffer, 2 µL RNAs free water, 5 μL of each 2 mM of deoxynucleotide triphosphate, 5 µL of Dream Taq DNA polymerase, 2 µL of 5 pm/µL Primer ILT (F: 5′ ACT GAT AGC TTT TCG TAC AGC ACG 3′ and R: 5′-CAT-CGG-GAC-ATT-CTC-CAG-GTA-GCA-3), and 3 µL template DNA and resulted in a 688 bp amplicon of the ICP4 gene fragment [32]. Thermal cycling conditions included an initial denaturation at 94 °C for 3 min, followed by 35 cycles of a three-step amplification protocol (denaturation at 94 °C for 30 s, annealing at 60 °C for 45 s, and elongation at 72 °C for 1:50 s), and finally one cycle of elongation at 72 °C for 10 min. PCR products were analyzed by 1.5% (*w*/*v*) agarose electrophoresis gel and visualized using a UV-lamp camera.

### 2.4. Statistical Analysis

Descriptive statistics were employed to summarize the study variables. Binary logistic regression was used to identify the potential risk factor for the ILTV. A first univariate logistic regression analysis was used, and those factors with a *p*-value < 0.25 were subjected to multivariable logistic regression. Odds ratios at a 95% confidence interval were used to express the strength of the association of the factors with the occurrence of the disease. Moreover, Hosmer–Lemeshow goodness of fit test was used to check the model’s adequacy. In the final model, a *p*-value of less than 0.05 with a 95% confidence interval (CI) was used to declare the associated factors. All the statistical analyses were performed by SPSS version 28 statistical software.

## 3. Results

### 3.1. Seroprevalence and Associated Risk Factors of Infectious Laryngotracheitis Virus

In the current study, 64 out of 240 blood samples tested were positive for ILTV-specific antibodies. The overall individual seroprevalence was 26.7% (ranging from 15.8 to 36.9% among different farms), and all the sampled poultry farms resulted positive (Table 1). Univariate analysis (chi-square) was used to investigate the influence of individual and managerial risk factors on ILTV seroprevalence. Several factors were positively associated with higher seroprevalence rates, including breed (local) and type of farm (backyard). Moreover, a higher prevalence was found in chickens raised on farms that introduced animals from other farms. Production purpose, age, sex, and farm size did not affect the ILTV seroprevalence. Variables with a *p*-value lower than 0.25 from the univariable analysis were included in the final multivariable logistic model based on a stepwise backward elimination procedure (Table 2). Backyard chickens were 1.464 times more likely to be affected by ILTV than commercial chickens (Table 3). For a unit increase in the number of introduced chickens, the odds of being affected by ILTV were increased by 1.52. The model has a good fit since the Hosmer and Lemeshow tests could not reject the hypothesis of model appropriateness with a value of *p* = 0.95.

### 3.2. Isolation and Molecular Detection of Infectious Laryngotracheitis Virus

**Figure 2:**
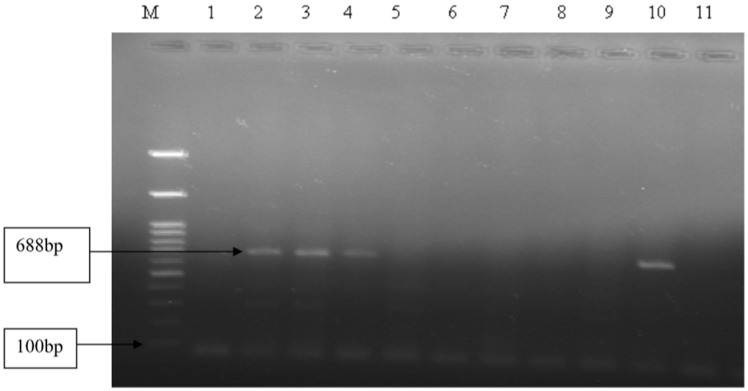
PCR amplification of 688 bp fragment of the ICP4 gene from ILTV-infected field samples. (M = 100-bp DNA ladder; 1 = Positive Control; 2, 3, 4, and 10 = field sample isolates showing ILTV-specific 688 bp products; 11 = Negative control).

Out of 15 ILTV infection-suspected samples that were inoculated onto the CAMs of 10-day-old embryonated SPF eggs via three consecutive passages, only six samples killed the embryo that showed white pock lesions on the CAM of the embryonated SPF egg (Table S1). Further analysis of molecular detections revealed that out of the 15 DNA samples tested, 4 (26.7%) were positive for ILTV (Table S2). Notably, PCR amplification produced a band of 688 bp in three tracheal swabs and one oropharyngeal swab sample (Figure 2). The positive samples were collected from layers and exotic-breed chickens.

## 4. Discussion

The overall seroprevalence of ILTV infection obtained from the present study across the eight kebeles was 26.7%. All sampled farms had positive animals, demonstrating the widespread diffusion of this pathogen in the studied area. This prevalence was similar to the finding of Baksi [33], who reported a 26.77% prevalence in India. However, the current finding was lower than that of the findings of Birhan et al. [23], Salhi et al. [34], Roba et al. [22], Mijanur et al. [35], Jahan et al. [36], and Shaza et al. [37], who reported a prevalence of 59.1% in Northwestern Ethiopia, 56.25% in Algeria, 54.7% in the Oromia region’s Ada’a districts, and 81.47%, 92.28%, and 96.7% in Bangladesh, respectively. Some of the above-stated countries use immunization against this virus; therefore, the antibodies found could possibly be vaccine-related. On the other hand, lower exposure rates were found in Central and South Ethiopia (19.4%), Ecuador (0.19%), Finland (12%), Bangladesh (0.4–17.33%), Iran (13%), and North Central Nigeria (1.2%) [21,38,39,40,41,42,43]. Furthermore, the differences observed between studies could be due not only to different epidemiological situations but also to the type of sampling carried out, the type of test used, the study period, etc.

The risk analysis revealed, as previously observed for other infections, that backyard chickens were more exposed to ILTV, most likely due to poorer biosecurity frequently observed on family farms and more interaction with wild animals [44]. On the other hand, backyard chickens may serve as a reservoir for wild birds due to their close association. The introduction of new chickens to the farm was another risk factor that was statistically associated with increased seroprevalence. This approach increases the chance of infection with any disease, particularly herpesvirus infection. In fact, apparently healthy animals with latent diseases might be placed on the farm, and when stressed, they facilitate the spread of the virus due to viral reactivation [45,46]. The absence of a difference in prevalence between layer and broiler chickens was unexpected, as, although all chickens are susceptible to the virus, the literature reports greater risks of infection between layers due to their longer productive lives [46].

The molecular prevalence (40%) obtained from testing suspected chickens was very similar to the seroprevalence rate. Previous studies performed in Ethiopia found molecular prevalence of 0% and 11% in 2017 and 2022, respectively [47,48]. In this case, the differences in prevalence derive above all from the type of matrix used rather than from the PCR protocol, which is rather standardized. In fact, experimental infections have established that 7 days after infection, the areas with the highest viral load are the conjunctiva, the trachea, the lungs, and the spleen [49]. Embryonated eggs inoculated with the field virus also have high viral loads, increasing the reliability of molecular methods.

Furthermore, the use of embryonated eggs (Table S1) also allows for the isolation of the virus so that it can be studied, sequenced, or used for the production of vaccines. Although the present study was performed on a limited number of samples, it demonstrated the circulation of ILTV in an area of Ethiopia where it had not yet been described and identified risk factors to take into consideration for the management of this infection. Further studies are necessary to fully understand the diffusion and impact that ILTV has on Ethiopian territory.

## 5. Conclusions

The present study demonstrated an overall ILTV seroprevalence of 26.7% and confirmed four ILTV-positive results in the backyard and commercial chickens of Robe Town, Southeastern Ethiopia. These data suggest that ILTV is widespread among backyard and commercial poultry farms in the study area. Introduced chickens and backyard production systems had increased seropositivity for ILTV. This study provides suggestions for control and biosecurity measures in the poultry farms to eradicate ILTV and represents a baseline for further study concerning ILTV in Ethiopia (such as sequencing of the viral genome to identify the strain).

## Figures and Tables

**Table 1:** Kebele (farm)-level seroprevalence of ILTV in chickens in the study district.

Kebele	No. Sample	Positive	Prevalence (%)	95% CI
Alage	33	12	36.4	22.19, 53.38
Basaso	19	4	21.1	8.51, 43.33
Bole	57	11	19.3	11.13, 31.34
Robe/01	19	3	15.8	5.52, 37.57
Robe/02	19	6	31.6	15.36, 53.99
Robe/03	27	7	25.9	13.17, 44.68
Robe/04	20	5	25.0	11.19, 46.87
Shallo	46	16	34.8	22.68, 49.23
Total	240	64	26.7	21.47, 32.6

**Table 2:** Univariable analysis for the occurrence of ILTV in chickens and its potential risk factors.

Factor	Examined (*n*)	Positive	Proportion (%)	95%CI	*p*-Value
Breed					
Exotic	226	56	24.78	9.15–30.41	
Local	14	8	57.14	31.22–83.07	**0.008**
Sex					
Male	46	9	19.57	8.10–31.03	
Female	194	55	35.71	15–43.28	0.22
Purpose					
Broiler	47	10	21.28	9.58–32.98	
Layer	193	54	27.98	21.65–34.31	0.35
Age					
>20 weeks	193	55	28.5	22.13–34.87	
8–20 weeks	47	9	19.5	7.9–30.4	0.19
Farm type					
Backyard	70	31	44.29	32.65–55.92	
Commercial	170	33	19.4	13.47–25.36	**<0.0001**
Chicken introduction					
No	17	13	76.47	53–90	
Yes	223	51	22.87	18–29	**<0.0001**

Bold *p*-values are significant ones.

**Table 3:** Multiple variable logistic analysis for the occurrence of ILTV and its potential risk factors.

Variable	*n*	Positive	Proportion (%)	AOR (95% CI)	*p*-Value
Chicken introduction					
No	17	13	76.47		
Yes	223	51	22.87	6.79 (2, 23.1)	**0.002**
Farm type					
Backyard	70	31	44.29		
Commercial	170	33	19.4	2.3 (1.19, 4.46)	**0.013**

Bold *p*-values are significant ones; AOR: Adjusted odds ratio.

## Data Availability

All data regarding this paper are available in this publication. However, further information can be requested from the corresponding author.

## Supplementary Materials

The following supporting information can be downloaded at: https://www.mdpi.com/article/10.3390/ani14223227/s1. Table S1: Isolation of ILTV from the field suspected samples using 10 days old embryonated SPF eggs for three consecutive passages; Table S2: Detail description of the active case with results.
