# Expression of Concern: Abebe et al. Serological and Molecular Investigation of Infectious Laryngotracheitis Virus in Chickens from Robe Town, Southeastern Ethiopia. *Animals* 2024, *14*, 3227

**DOI:** 10.3390/ani16050796

**Published:** 2026-03-04

**Authors:** Animals Editorial Office

**Affiliations:** MDPI, Grosspeteranlage 5, 4052 Basel, Switzerland; animals@mdpi.com

With this notice, the *Animals* Editorial Office wishes to alert readers to concerns related to this article [1]. Following publication, concerns were raised regarding the ethical oversight, authorship, and provenance of the data contained within the article.

The authors of this publication, Hawassa University, and the National Veterinary Institute (NVI) have been contacted for further clarification. The investigation is being coordinated by the Editorial Office with the involvement of the Editorial Board. Once this process is complete, the Editorial Office will provide an update on this situation and announce any post-publication modification deemed to be necessary, as per MDPI’s Updating Published Papers policy (https://www.mdpi.com/ethics#_bookmark30).
