# Supplementary material for: Serological and Molecular Investigation of Infectious Laryngotracheitis Virus in Chickens from Robe Town, Southeastern Ethiopia
Source: Animals (Basel). 2024 Nov 11;14(22):3227. doi: 10.3390/ani14223227 (PMC11591540; doi:10.3390/ani14223227)
Supplement: Supplementary file 1 [file animals-14-03227-s001.zip › animals-3227955-supplementary.pdf]

Table S1. Isolation of ILTV from the field suspected samples using 10 days old embryonated SPF eggs for three consecutive passages

Sample (Swab)	Con. Spin (CAM)	Passage number and mortality across different time														
		Passage 1					Passage 2					Passage 3				
		24hrs	48hrs	72hrs	96hrs	120hrs	Same time order					Same order time				
Trachea	0.2ml	0	2/4	0	0	0	0	4/4	0	0	0	0	4/4	0	1/4	0
Trachea	0.2ml	0	2/4	1/4	¼	0	0	4/4	0	0	0	0	4/4	0	0	0
Orophar	0.2ml	0	1/4	3/4	0	0	0	4/4	0	0	0	0	4/4	0	0	0
Orophar	0.2ml	0	2/4	3/4	0	0	0	4/4	0	0	0	0	4/4	0	0	0
Trachea	0.2ml	0	3/4	1/4	0	0	0	3/4	0	0	0	0	4/4	0	0	0
Orphar	0.2ml	0	2/4	2/4	0	0	0	3/4	¼	0	0	0	3/4	0	1/4	0

Note: con= concentration of sample inoculated, CAM= chorioallantoic membrane, oropharyngeal swab, trachealswab.

Table S2: Detail description of the active case with results

Case	Location	Month	Production purpose	Sample type	VI test	PCR test
1	Robe/01	April	Layer	Oropharyngeal swab	Neg	Neg
2	Robe/04	April	Layer	Tracheal swab	Positive	Positive
3	Robe/03	May	Layer	Tracheal swab	Positive	Positive
4	Bole	April	Layer	Oropharyngeal swab	Positive	Positive
5	Alage	February	Layer	Tracheal swab	Neg	Positive
6	Bole	May	Layer	Oropharyngeal swab	Positive	Neg
7	Bole	April	Layer	Tracheal swab	Neg	Neg
8	Bole	April	Layer	Oropharyngeal swab	Positive	Neg
9	Shallo	February	Layer	Tracheal swab	Nega	Neg
10	Robe/03	May	Layer	Oropharyngeal swab	Positive	Positive
11	Shallo	February	Layer	Tracheal swab	Neg	Neg
12	Alage	February	Layer	Oropharyngeal swab	Neg	Neg
13	Alage	February	Layer	Tracheal swab	Neg	Neg
14	Shallo	February	Layer	Tracheal swab	Neg	Neg
15	Robe/04	April	Layer	Tracheal tissue	Neg	Neg

Note: Neg: negative, VI: virus isolation, PCR: polymerase chain reaction
